# Toward Generalized Concern: The Development of Compassion and Links to Kind Orientations

**DOI:** 10.1177/07435584211007840

**Published:** 2021-04-24

**Authors:** Joanna Peplak, Tina Malti

**Affiliations:** 1 University of California, USA; 2 University of Toronto, Canada

**Keywords:** ethical development, compassion, prosocial development, mixed methods

## Abstract

Compassion underlies kindness and as such, is important for creating harmonious societies. We examined children and adolescents’ personal experiences of compassion and then how youth with different compassion profiles differed in their kindness (i.e., dispositional sympathy and prosocial behavior). An ethnically diverse sample of 8-, 11-, and 15-year-olds (*N* = 32; 66% girls) provided narratives of times they felt compassion. Next, in another diverse sample of 7-, 11-, and 15-year-olds (*N* = 168; 49% girls), we assessed youths’ potential for global compassion (i.e., compassion that transcends intergroup boundaries) using a novel interview procedure. We also collected self- and caregiver-reports of dispositional sympathy and prosocial behavior. Youths’ narratives revealed that youth often experienced compassion toward peers and relatives following both physical and psychological sufferance and often mentioned responding to the suffering other with helping behavior. On average, youth reported moderate levels of global compassion (i.e., compassion toward a suffering victimizer) and developmental trends revealed that 15-year-olds reported lower feelings of compassion than 11-year-olds. Next, latent profile analysis showed that compassion-oriented youth (i.e., youth who displayed moderate-high levels of global compassion) were rated as more prosocial than non-compassion-oriented youth (i.e., those who displayed low levels of global compassion). We discuss findings in relation to theory and research on the development of kindness in general and in intergroup contexts.

In turbulent times characterized by conflict, divide, and global crises (e.g., the COVID-19 pandemic), expanding our range of concern for others to extend across intergroup boundaries may be important for motivating reparation, cooperation, and harmony. Nurturing compassion may be particularly necessary to conquer conflict and hardship. *Compassion* is a foundational component of kindness (Malti, 2020; Schopenhauer, 1915) and is defined as a feeling of concern for a suffering other that involves a desire to alleviate their pain (Goetz et al., 2010). Recently, due to increased globalization and diversification, the importance of *global compassion* (i.e., compassion that is felt toward others regardless of their status, intergroup membership, or circumstance) has been highlighted. Global compassion is theorized to be fundamental in the development of a universal prosocial orientation and may motivate kindness that extends beyond one’s own group (Ekman & Ekman, 2017). Despite a consensus among scientists that compassion has intra- and interindividual benefits (Goetz et al., 2010), surprising little effort has been placed into understanding youths’ experiences of compassion (Spinrad & Eisenberg, 2017).

The overarching goals of this study were to first explore children’s and adolescents’ narratives of times they felt compassion for someone. Then, using knowledge acquired from these narratives, we investigated how youths’ global compassion may develop by assessing their feelings of compassion toward a victimizer—an out-group member that harbors feelings of dislike, dissimilarity, and repulsion (Rosenbaum, 1986). Finally, we investigated how children and adolescents with various capacities for global compassion differed in their kindness. We examined these research questions in youth ages 7–8, 11, and 15 years because there are important shifts in social-emotional skills from middle childhood to adolescence that may influence one’s ability to feel compassion for others. For example, children’s capacity to feel other-oriented concern increases until late childhood, their ability to take the perspective of others and engage in moral reasoning advances through adolescence (Eisenberg et al., 2015), and youth are better able to integrate and balance multiple perspectives in complex social situations from childhood to adolescence (Killen & Malti, 2015).

## Conceptualization of Compassion

Compassion falls within the “other-oriented emotion” family along with empathy and sympathy (see Table 1 for summary of key differences between compassion and related constructs). Recent theories and research have highlighted various antecedents that are likely involved in experiences of compassion (Goetz et al., 2010; Spinrad & Eisenberg, 2017). One antecedent that may specifically prompt compassion is another’s physical and/or psychological suffering (Stellar et al., 2020). Aristotle identified suffering to be the primary antecedent of compassion and laboratory studies have used instances of physical sufferance (e.g., illness, disability, physical pain; Eisenberg et al., 1988; Oveis et al., 2010; Stellar & Keltner, 2014) to reliably evoke compassion in children and adults. Nevertheless, we know little about the contextual factors that may prompt compassion. In attempt to address this research gap, we first gathered youths’ narratives of a time they felt compassion for another and assessed when, toward whom, and how they responded in those contexts.

**Table 1.** Comparing Compassion with Related Constructs.

Concept	Definition	Relation to compassion
Empathy	An emotional capacity that stems from the comprehension of another’s emotional state or condition and is similar to what the other is feeling (Eisenberg, 2000).	*Similarities*: Both are implicated in situations involving the harm of another. *Differences*: Empathy is not always other-oriented, whereas compassion is. Empathy developmentally precedes compassion and involves less cognitive complexity than compassion, is pan-affective, and is more likely to lead to personal distress. Compassion and empathy activate different brain regions and compassion has been related to positive subjective experiences (see Klimecki et al., 2014).
Sympathy	A feeling of sorrow or concern for a needy other (Eisenberg, 2000).	*Similarities*: Both are other-oriented emotions that involve a feeling of sorrow or concern for a less fortunate other. *Differences*: Sympathy may be more impacted by situational factors and does not necessarily involve components of tolerance and a desire for help.
Personal Distress	A self-focused, aversive, affective reaction to the apprehension of another’s emotion (e.g. discomfort or anxiety; Eisenberg, 2000).	*Similarities*: Both occur in response to the suffering of another. *Differences*: Personal distress is a self-focused and dysregulated response while compassion is other-oriented and regulated. Negative relations (or no relations) between personal distress and prosocial behavior has been found whereas compassion is positively related to prosocial behavior.
Perspective Taking	The ability to understand another’s thoughts, feelings, or situation (Moll et al., 2013)	*Similarities*: Both perspective taking and compassion lead to prosocial behaviors because understanding of, and affective responsiveness to, the needs of others is purported to facilitate actions that aim to relieve their distress. *Differences*: Perspective taking does not include an affective component.
Kind Orientation	An umbrella term that reflects one’s propensity to experience emotions, thoughts, and express behaviors that reflect fairness, care, and a concern for the welfare of others (Malti, 2020).	*Similarities*: Compassion may be considered a type of kind orientation as it is other-oriented and reflects one’s concern for the welfare of others. *Differences*: Kind orientations reflect one’s *general* drive to care for others, which does not exclusively occur in response to their suffering (e.g., generosity).

### Components of Compassion

Compassion is conceptualized as a multicomponent affective response that involves (a) feelings of concern for a suffering other or group, (b) tolerance, and (c) a desire to help the sufferer (Goetz et al., 2010; Malti & Latzko, 2017; Strauss et al., 2016).

Compassion includes a prominent affective component that is characterized by feelings of sorrow or concern for a suffering other (akin to sympathy; Stellar & Keltner, 2014). Tolerance is an attitudinal component that allows an individual to endure and regulate one’s thoughts and feelings when confronted with another’s suffering (also termed distress tolerance) and overcome any negative dispositions toward the other to make way for feelings of compassion (also termed non-judgment; Feldman & Kuyken, 2011; Gilbert, 2010). Finally, compassion galvanizes a desire to alleviate the pain of the suffering other (Goetz et al., 2010)—a motivation that is likely catalyzed by one’s feelings of concern and maintained via tolerance. Indeed, children are more likely to engage in helping another if they are prompted to experience high levels of concern for them (Svetlova et al., 2010; Vaish et al., 2009); however, if concern is not coupled with tolerance, one’s desire to help may be thwarted by situational factors such as the costliness of helping, the intergroup status of the suffering other, and/or one’s mood (Spinrad & Eisenberg, 2017).

### Types of Compassion: Global Versus Familial

There is consensus among scholars that care is often partial—we attend to and care for those who are closest to us (Frakes, 2010). To address the role of partiality in feelings of compassion, Ekman and Ekman (2017) have recently outlined unique subtypes of compassion. The two types that are relevant to the current investigation are familial compassion and global compassion. *Familial compassion* is felt toward kin or familiar others in response to their suffering and is thought to be readily expressed. This is reflected in children’s tendencies to respond with higher levels of kind emotions, moral judgments, and prosocial behavior toward familiar, in-group members (e.g., individuals of the same age and gender, and family/friends) than unfamiliar, out-group members (e.g., disliked others, strangers; e.g. Eisenberg, 1983; Eisenberg & Spinrad, 2014; Weller & Lagattuta, 2013). *Global compassion* is the feeling of concern and desire to alleviate the suffering (proximal or distal) of anyone, regardless of their background or intergroup membership (Ekman & Ekman, 2017). This type of compassion involves widening one’s sphere of concern beyond family and friends to strangers and enemies (Ekman & Ekman, 2017; Frakes, 2010). To feel global compassion, one must recognize common human desires, notably that everyone wants to avoid suffering (Frakes, 2010). In this study, to garner knowledge on how youth may experience global compassion, we assessed their feelings of concern and desire to help an out-group peer—specifically, a peer who had previously harmed them (i.e., a victimizer). Because victimizers cause harm, they are often disliked and considered part of one’s out-group (according to the dissimilarity-repulsion principle; Chen & Kenrick, 2002). We chose to avoid more salient intergroup categories (e.g., race/ethnicity and gender) to limit the role of any complex pre-formed biases and prejudices that accompany these groups categories so that we could isolate situational intergroup sentiments; nevertheless, we consider these categories important to consider in future research.

## Development of Compassion

Our capacity for care is rooted in both biological foundations and affiliative experiences which allow us to experience empathic responses beginning in the first year of life (Davidov et al., 2013; Gilbert, 2015; Zahn-Waxler et al., 1992). In the second year of life, when children acquire the ability to differentiate the self from the other, they begin to express concern for others and show prosocial responding toward others in need (Eisenberg, 2000). Perspective taking skills (i.e., skills that reflect one’s ability to look beyond one’s point of view and understand another’s thoughts and feelings), particularly affective perspective taking (i.e., imagining or inferring what the other person is feeling; Vaish et al., 2009), are fundamental for fostering compassion such that they allow youth to understand another’s emotions and circumstances (Malti et al., 2013). These skills begin to emerge in the preschool years (Moll et al., 2013) and continue to unfold across development into adolescence (Selman, 1980).

Despite the lack of research on the development of compassion, particularly global compassion, researchers have demonstrated that in toddlerhood, children are able to experience compassion-related responding (e.g., feeling concern and empathic helping in response to minor physical harm) in straightforward contexts involving the harm of their caregivers or familiar adults (e.g., Spinrad & Stifter, 2006; Svetlova et al., 2010; van der Mark et al., 2002), but show difficulty extending their compassion toward strangers or those they do not like (Eisenberg, 1983; Sierksma et al., 2015; Weller & Lagattuta, 2013). It is possible that, with the development of regulation and perspective taking skills, children develop tolerance, which then allows them to extend their compassion beyond their immediate group. As research by Eisenberg (1983) shows, children become less selective with age in determining who among various recipients (e.g., friends, kin, disliked others) should be helped when in need.

However, it is also possible that children may steer further away from global compassion across development. Accordingly, although trends in the research show that children’s sympathy for others in need and their prosocial behavior generally increases from childhood to adolescence (see Eisenberg et al., 2015), some research demonstrates that children become more selective with their kindness (Hay & Cook, 2007; Kanacri et al., 2013), conserving their prosociality for in-group members (see Eisenberg & Spinrad, 2014; see Malti, Gummerum, et al., 2016; Sierksma et al., 2015; Weller & Lagattuta, 2013). In attempt to clarify discrepancies in developmental findings related to global compassion, we explored developmental differences in youths’ feelings of compassion in response to harm to victimizers.

## Profiles of Global Compassion: Individual Differences in Kind Orientations

Research has recently highlighted the importance of child-centered approaches to identify subgroups of individuals within a sample that share similar characteristics (Newton et al., 2016). Here, it was assessed whether there are subgroups of children who have different capacities for global compassion. Then, to assess why these groups may differ, we examined profile differences in core kindness orientations. We focus on dispositional sympathy (i.e., defined as one’s general tendency to feel concern for needy others) and dispositional prosocial behavior (i.e., defined as one’s general tendency to engage in behaviors intended to help others) because both are conceptually related to compassion and considered central components of kindness (Eisenberg et al., 2015; Malti, 2020).

### Compassion and Sympathy

Dispositional sympathy is conceptualized as a characteristic that reflects altruistic values and one’s general abilty to experience concern for others (Eisenberg et al., 1989). Although situational and dispositional sympathy are related, individuals may vary in their propensity to react sympathetically despite potentially high levels of situational sympathy (Maibom, 2012). Examining whether youth who experience high levels of global compassion also experience high levels of dispositional sympathy may provide insight into whether compassion may be driven by an advanced capacity for concern.

### Compassion and Prosocial Behavior

Prosocial behavior is voluntary behavior that is intended to benefit another and is often motivated by other-oriented emotions and internalized altruistic values (Eisenberg et al., 2015). Similar to dispositional sympathy, dispositional prosocial behavior reflects how prosocial an individual is in general. Little is known whether compassion may be a motivational feature underlying general tendencies to engage in prosocial behavior across time and contexts. Here, we examined whether youth with different profiles of compassion differed in their dispositional prosocial behavior to understand whether compassion may have benefits for youths’ general prosociality (or vice versa).

## The Present Study

We employed a mixed-method approach to assess three aims. First, we explored children and adolescents’ (aged 8, 11, and 15 years) experiences of compassion via narratives—an approach that emphasizes the interpretive power of personal stories and provides a window into how youth create meaning within their socio-moral experiences (Wainryb et al., 2005). Due to the exploratory and qualitative nature of this aim, we did not have specific hypotheses—rather, our goal was to distinguish various contextual facets of children’s experiences of compassion (e.g., the types of targets and the type of harm that precede compassion) and how youth express compassion.

Next, using knowledge generated from youths’ compassion narratives, we developed and employed a novel interview procedure to examine our second study goal which was to investigate whether and how children and adolescents (7-, 11-, and 15-year-olds) experience global compassion. Much of the previous work on children and adolescents has focused on employing questionnaires to assess youths’ compassion-related responding (e.g., sympathy) or other methods (e.g., facial and physiological responses to distressing videos) that only measure facets of compassion. Our vignette procedure extends this work by providing insight into youths’ situational responses within compassion-inducing scenarios. First, using a variable-centered approach, we assessed age-related differences in expressions of compassion. We hypothesized that youth of all ages would express moderate levels of global compassion as we expected that feeling compassion for a suffering victimizer would be challenging. Due to contrasting evidence showing both age-related declines and increases in compassion-related responding across childhood into adolescence, expected findings were unclear. However, we anticipated that competing social concerns (e.g., maintaining a high social status and positive in-group identity; Horn, 2003; LaFontana & Cillessen, 2010) would hinder adolescents’ (15-year-olds’) feelings of compassion, resulting in lower levels of compassion compared to younger children.

Next, using a child-centered approach, we aimed to identify whether there were different subgroups within our sample who differed in their ability to experience global compassion and whether these groups of children and adolescents would diverge in their dispositional sympathy and prosocial behavior. Because dispositional sympathy reflects one’s altruistic orientation and tendency to feel concern (Eisenberg et al., 1989), high levels of dispositional sympathy may be important for prompting global compassion in youth. Likewise, since prosocial behavior is often motivated by compassion, we hypothesized that youth who experienced global compassion would be more generous and less discriminate with their prosociality and thus have higher levels of dispositional prosocial behavior.

## Method

### Participants

Due to the phenomenological nature of narratives and based on data saturation, a small sample size was targeted for the qualitative portion of the study (Creswell, 1998; Guest et al., 2006). A community sample of 36 participants were recruited from a metropolitan region in Canada. Data from 4 participants were missing due to technical issues with audio recording. Thus, data from 32 participants (66% girls) aged 8 (*n* = 8, *M*_age_ = 8.44, *SD* = 0.38), 11 (*n* = 11; *M*_age_ = 11.65, *SD* = 0.47) and 15 years (*n* = 13; *M*_age_ = 15.61, *SD* = 0.39) were used. Participants were of diverse ethnic origins, including participants from European (44%), Asian (31%), Middle Eastern (3%), Caribbean and South American (3%), and other or multiple ethnic backgrounds (e.g., Eurasian, Canadian, 13%; 6% did not report their ethnic origin). As a proxy for family socioeconomic status, primary caregivers reported their highest completed level of education, such that 6% received an apprenticeship/trades diploma, 72% received a university/college degree, and 22% earned a postgraduate degree.

Next, a community sample of 168 7- (*n* = 59; *M*_age_ = 7.48, *SD* = 0.28; 55% girls), 11- (*n* = 53; *M*_age_ = 11.37, *SD* = 0.29; 49% girls), and 15-year-olds (*n* = 56; *M*_age_ = 15.39, *SD* = 0.19; 54% girls), as well as their primary caregivers, participated in the quantitative portion of the study. The sample size was chosen based on G* Power 3.1 analyses to detect medium effects (*f*^2^ = .15) when conducting multiple regression to accommodate our mixed-method approach (Erdfelder et al., 1996) and is sufficient for latent profile analysis (LPA) with a small number of items and few anticipated classes (Tein et al., 2013). Similar to the above sample, our participants were ethnically diverse, including participants from European (40%), Asian (18%), Caribbean and South American (11%), Indigenous (2%), African, (1%), and other or multiple ethnic backgrounds (e.g., Canadian-Caribbean, Indian-Scottish, Jewish, African-Canadian; 21%; 7% did not report their ethnic origin). This is representative of the city from which the data were collected (Statistics Canada, 2016). Caregivers reported their highest level of education: 69% were university/college graduates, 21% were postgraduates, and 5% were high school graduates. Five percent of caregivers did not report their education.

### Procedure

The study was approved by the Research Ethics Board of the researchers’ institution. Informed written consent was obtained from the caregiver and oral assent from the children and adolescents for both the qualitative and quantitative parts of the study. Each participant and their primary caregiver visited the research laboratory for a one-time session. Trained psychology students interviewed children and adolescents in a private testing room for approximately 15 to 30 minutes. The session was video recorded for data analysis purposes. For the qualitative portion of the study, caregivers completed a questionnaire regarding their demographic information. For the quantitative portion, caregivers reported on their children’s social-emotional development in addition to their demographics. At the end of the session, children and adolescents were given a book of their choice for participating.

### Measures

#### Compassion narratives

Participants were asked to recount times they felt compassion for someone (i.e., “Tell me about a time you felt compassion. Pick a time you remember well and tell me everything you remember about that time.”) This procedure was adapted from developmental narrative research (Wainryb et al., 2005). When participants stated that they did not understand compassion or asked for clarification (84% of participants), the question was rephrased to provide the respective definition of compassion. The prompt was: “Tell me about a time you felt concern for someone because they were suffering and you wanted to help them” (definition from Goetz et al., 2010). If participants did not ask for the definition of compassion, the interviewer monitored their response to ensure they were providing appropriate responses that reflected an understanding of the term.

##### Coding

Coding categories, descriptions, and prototypical examples can be found in Table 2. The coding scheme was developed based on ours and others’ related research (codebook thematic analysis approach; Malti et al., 2009) and themes were refined or added through inductive data engagement (Braun & Clarke, 2020). Two coders consensus coded all narratives. Narrative responses were coded for the target of compassion (peers, relatives, and unknown others), type of harm (physical, psychological, general condition), and prosocial responding (helping, comforting, showing concern). One category was coded for each domain (i.e., target, type of harm, prosocial response).

**Table 2.** Coding Categories and Examples of Themes Within Youths’ Compassion Narratives.

Category	Description	Example
Target		
Peers	Friends or acquaintances of a similar age.	Friends, classmates, schoolmates.
Relatives	Individuals in the family such as siblings or grandparents.	Siblings, parents, grandparents.
Unknown others	Individuals that the children do not personally know.	The homeless, the less fortunate, individuals from different countries
Type of Suffering		
Physical	Themes of physical pain, injury, or illness.	“[. . . ] sister was sick from removing her tonsils.”
Psychological	Themes of failure, relational harm or mental health issues.	“[. . . ] a friend [. . . ] was feeling really depressed.”
General state	Themes of suffering due to the political, social, and economic state of the individuals’ environment.	“[. . . ] people [who are] paid no wages or like very little and they’re put in very bad conditions.”
Prosocial Response		
Helping	Actively trying or wanting to improve the target’s problem.	“[. . . ] I helped him out by studying with him.”
Comforting	Consoling the target or showing concern for them through facial expressions, physical comfort, and/or condolences.	“[. . . ] trying to talk to her a lot and just being beside her.”
No action	No mention of prosocial response.	—

#### Global compassion vignettes

Six vignettes were developed based on previously validated vignettes on morally relevant emotions (Malti et al., 2009) to measure youths’ global compassion. Because we found that most compassion narratives were directed at peers and involved both physical and psychological harm (see results from compassion narratives below), each vignette depicted a peer who was age- and gender-matched to the participant and showed a variety of instances of physical and emotion harm. Each story began with the protagonist hypothetically causing the participant harm, and later, the protagonist was harmed in a similar way by a third-party hypothetical peer. An example vignette is:

Imagine you are beaten up by two boys/girls. You don’t know any of the boys/girls and start crying. A few days later, you see one of the two boys/girls who picked on you being beat up by two other boys/girls.

The other vignettes were similarly structured and involved other types of harm such as exclusion, prosocial omission, and verbal aggression. A suffering victimizer was chosen as the target of children’s global compassion because (a) victimizers are considered part of the out-group and feelings of compassion toward victimizers likely reflects one’s ability to extend their concern beyond one’s own group, (b) victimizers likely incite dislike, anger and/or hatred in children—feelings that reinforce intergroup distinctions and require tolerance to overcome, (c) children have likely encountered a victimizer (or someone they do not like) experience harm in their daily life, thus rendering their emotional expressions to our vignettes authentic and (d) the Dalai Lama (2002) argues that “for a practitioner of love and compassion, an enemy is one of the most important teachers. Without an enemy you cannot practice tolerance, and without tolerance you cannot build a sound basis of compassion” (p. 75). Vignettes were piloted (*N* = 10) prior to study commencement to ensure developmental appropriateness and comprehension. Minor changes in wording were subsequently employed.

##### Concern

After each vignette, participants were asked to rate how sorry they felt for the protagonist (i.e., victimizer) on a 4-point Likert-type scale from 1 = *not sorry* to 4 = *sorry*. Cronbach’s alpha between stories was good (α = .70). We then aggregated compassionate concern across our six vignettes for subsequent analyses.

##### Desire to help

Youths’ desire to help the suffering victimizer was examined using the dictator game (Kahneman et al., 1986). Participants were provided with six chocolate coins following each vignette and asked whether they wanted to share (or not) with the suffering victimizer by putting the coins they wanted to share in a box that belonged to the victimizer. Participants were told that the coins they gave away would go to the victimizer and the coins they kept would be given to them at the end of the study. The interviewer looked away as the participant divided their coins. We used sharing as a proxy for participants’ desire to help to assess real rather than hypothetical intentions to help alleviate the pain of the victimizer. We believe that children’s actual sharing of goods would involve less desirability bias than self-reports about intentions. Sharing resources likely showcases one’s desire to increase the happiness and decrease the negative affect of the suffering other. A mean score of the amount of coins participants shared across vignettes was calculated for subsequent analyses (a value between 0 and 6); Cronbach’s alpha was good (α = .75).

##### Preference (control)

We controlled for preference (i.e., how much participants enjoy chocolate coins; 1 = *not much*, 2 = *somewhat*, 3 = *very much*) when examining developmental differences in participants’ desire to help.

#### Kind orientations

##### Dispositional sympathy

Youth- and caregiver-reports of dispositional sympathy were collected using 4 items each from a previously validated scale (Child and Teacher Sympathy Scale; see Eisenberg et al., 1996). An example youth﻿-report item is “I often feel sorry for other children/peers who are sad or in trouble.” Youth rated each item on a 3-point Likert-type scale (1 = *not like me*, 2 = *sort of like* me, or 3 = *really like me*). Cronbach’s alpha was acceptable (α = .62). An example item for caregiver-reports is “my child usually feels sympathy for other children who are upset or sad.” Caregivers reported how true each item was of their child on a 6-point Likert-type scale (1 = *not at all true*, 6 = *always true*). Cronbach’s alpha was good (α = .83).

##### Dispositional prosocial behavior

Youths’ dispositional prosocial behavior was measured using the Prosocial Behavior Subscale from the Strengths and Difficulties Questionnaire (Goodman, 1997). Caregivers rated how true each of the 5 items was of their child on a 6-point Likert-type scale from 1 = *not at all true* to 6 = *always true*. An example item is “my child often volunteers to help others.” Cronbach’s alpha was acceptable (α = .60; see Zuffianò et al., 2015 for a similar alpha).

### Data Analytic Approach

Frequencies and patterns of themes in youths’ compassion narratives were assessed to examine the various components involved in youths’ experiences of compassion. Frequencies of themes within each category of interest (i.e., target of compassion, type of harm experienced by the target, and responses to the target) were calculated and patterns were assessed to inform the quantitative portion of the study.

Next, to more systematically assess youths’ compassion and garner insight into global compassion, we first ran descriptive statistics and bivariate correlations to examine patterns and zero-order relations between our study variables. We then conducted two analyses of covariance (ANCOVAs) to test developmental differences in our compassion variables (i.e., concern and desire to help). Next, LPA (Lanza et al., 2003) was employed using M*plus* 8.0 (Muthén & Muthén, 1998–2017) to derive categorical latent variables that represent groups of youth who share similar compassion profiles based on their compassionate concern and desire to help. LPA moves beyond variable-centered approaches by testing how a *combination* of variables differ across individuals and in doing so, uses model-based clustering (Newton et al., 2016). Models with 1–4 latent classes were tested iteratively to determine which model best fit the data. The best-fitting model was determined by considering the interpretability of the results as well as comparing the Bayesian information criteria (BIC), Akaike information criteria (AIC), entropy, and the Lo–Mendell–Rubin Adjusted LTR Test (LMRT; Nylund et al., 2007). Class size was also considered when determining the optimal number of profiles (Hipp & Bauer, 2006). After selecting the best-fitting solution, our outcome variables (sympathy and prosocial behavior) were regressed on our control variables (age, gender and caregiver education) and then we conducted *t*-tests to evaluate differences in our outcome variables as a function of profile membership.

## Results

### Compassion Narratives

**Figure 1.**
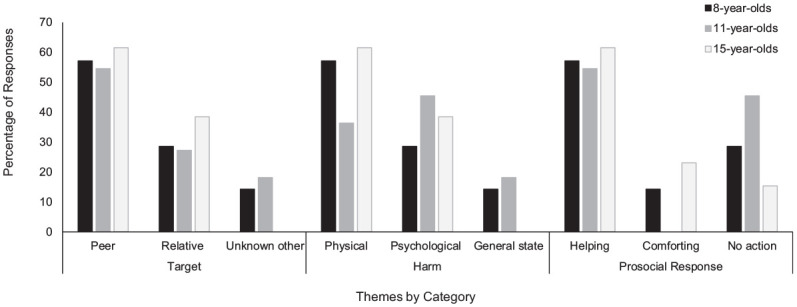
Narrative themes by age group.

Our findings revealed that the majority of youth mentioned peers (56%) or relatives (31%) as the targets of their compassion, and only a small portion (9%) mentioned unknown others or groups (e.g., homeless people). The types of suffering or harm that were mentioned varied between the narratives, with physical suffering being the most prevalent (44%) followed by psychological suffering (38%) and general-state sufferance (9%). Finally, participants most often mentioned helping or wanting to help the suffering other (50%) followed by comforting (12%), and a quarter of the participants did not spontaneously mention any type of prosocial response (25%). Figure 1 displays themes mentioned by age group. Among youth who understood compassion and did not require the definition before providing their narrative (5/32 participants; two 11-year-olds and three 15-year-olds), findings revealed that all participants mentioned peers as their targets of compassion, all but one (4/5) reported psychological instances of harm suffering, and all but one (4/5) mentioned helping or wanting to help the target. Below is an example of a complete compassion narrative from a 15-year-old girl about her relative reflecting themes of physical suffering and helping:

One time my grandma was really sick and I was concerned about her. She had trouble with her breathing and she would get really bad anxiety. I felt concerned for her and I wanted her to get better and I wanted to help her, so I tried to be nice or get her water.

### The Development of Global Compassion

#### Descriptive statistics

Table 3 provides means and standard deviations of all variables by age group. Table 4 provides correlations of all study variables. As expected, compassionate concern was strongly and positively related to desire to help. Both of our compassion variables were positively related to child-reported sympathy and caregiver-reported prosocial behavior, but they were not correlated with caregiver-reported sympathy. Furthermore, caregiver-reported sympathy and prosocial behavior were positively correlated. Girls scored higher than boys on compassionate concern, desire to help, sympathy, and prosocial behavior. This is in line with previous research showing gender effects in sympathy and prosocial behavior favoring girls (see Eisenberg et al., 2015 for review) and may be due to divergent gender role and socialization experiences of girls versus boys.

**Table 3.** Means and Standard Deviations of Study Variables by Age Group.

Variable	7-year-olds (*n* = 59)		11-year-olds (*n* = 53)		15-year-olds (*n* = 56)		Overall (*N* = 168)	
	*M*	*SD*	*M*	*SD*	*M*	*SD*	*M*	*SD*
Concern	2.84	0.61	2.89	0.53	2.61	0.55	2.78	0.58
Desire to help	2.50	0.94	2.76	0.84	2.41	0.92	2.55	0.91
YR sympathy	2.64	0.42	2.62	0.30	2.64	0.36	2.64	0.37
CR sympathy	4.50	0.66	5.17	0.60	4.99	0.64	5.05	0.64
CR prosocial behavior	5.10	0.55	5.38	0.47	5.18	0.53	5.21	0.53
Caregiver education	4.09	0.85	3.94	0.86	3.89	0.76	3.94	0.78

*Note.* Concern ratings ranged from 1 to 4. Desire to help ranged from 0 to 6. Child-reported sympathy scores ranged from 1 to 3. Parent reported ratings ranged from 1 to 6. Caregiver education ranged from 1 (elementary school education) to 5 (graduate school). YR = Youth-reported; CR = Caregiver-reported.

**Table 4.** Correlation Matrix of Study and Control Variables.

Variable	1	2	3	4	5	6	7	8
1. Concern	—							
2. Desire to help	.54***	—						
3. YR sympathy	.14^†^	.18*	—					
4. CR sympathy	.03	.09	.02	—				
5. CR prosocial behavior	.17*	.18*	.03	.68***	—			
6. Age	−.11	−.01	−.00	−.03	.06	—		
7. Gender	−.17*	−.16*	−.20*	−.25**	−.26**	.01	—	
8. Caregiver education	.05	−.11	−.05	−.04	.10	−.12	−.05	—

*Note.* Gender was coded −1 for girls and 1 for boys. YR = Youth-reported, CR = Caregiver-reported.

† *p* < .10. *p < .05. **p < .01. ***p < .001.

#### Developmental differences in global compassion

ANCOVAs were conducted to test age-related differences in concern and desire to help. After controlling for gender, the corrected model for compassionate concern was significant, *F*(2, 164) = 4.29, *p* < .05, η_p_^2^ = .05. Bonferroni post hoc comparisons revealed that 15-year-olds reported lower rates of concern compared to 11-year-olds (*M*_*diff*_ = −.29, *p* < .05), and marginally lower rates than 7-year-olds (*M*_*diff*_ = −.24, *p* < .10). After controlling for gender and preference (i.e., how much participants reported liking chocolate coins), there were no age-group differences in desire to help, *F*(2, 159) = 2.38, *p* < .10, η_p_^2^ = .03.

### Profiles of Global Compassion and Differences in Kind Orientations

**Figure 2.**
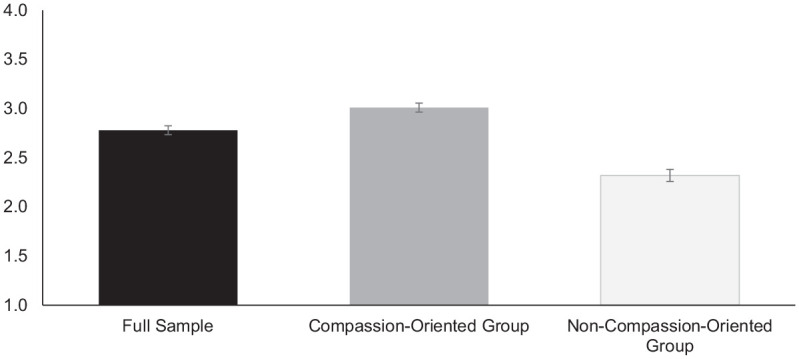
Means of concern in the full sample and in each latent class from the two-class model. *Note.* The scale for concern ranges from 1 to 4. Error bars display standard errors of the mean.

**Figure 3.**
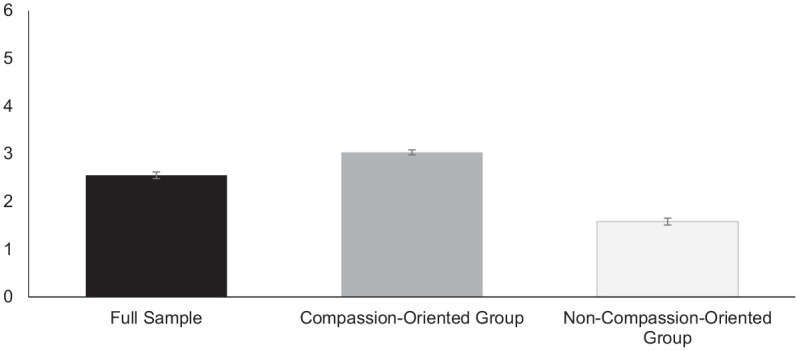
Means of desire to help in the full sample and in each latent class from the two-class model. *Note.* The scale for desire to help ranged from 0 to 6. Error bars display standard errors of the mean.

Comparisons of LPA model fit are presented in Table 5. Based on the fit indices and class sizes, the model with two profiles had the best fit (Lanza & Cooper, 2016). The two profiles were labeled “compassion-oriented” (*n* = 141) and “non-compassion-oriented” (*n* = 54) based on mean levels of concern and desire to help. As displayed in Figures 2 and 3, the compassion-oriented group had an above-average score on concern (*M* = 3.01) and desire to help (*M* = 3.03) compared to the overall sample (*M* = 2.78, *M* = 2.55, respectively), while the non-compassion-oriented group had lower than average scores on concern (*M* = 2.32) and desire to help (*M* = 1.58). When assessing age-group and gender differences in profile membership, we found that 11-year-olds were more likely to be in the compassion-oriented group than 7-year-olds, χ^2^(1) = 5.44, *p* < .05, and 15-year-olds, χ^2^(1) = 4.65, *p* < .05. There were no differences in profile membership between girls and boys.

**Table 5.** Fit Indices for Latent Class Analysis Models With One Through Five Latent Profiles.

Statistic	Number of profiles				
	1	2	3	4	5
Log Liklihood	−367.18	−338.89	−330.92	−324.29	−321.47
AIC	742.36	691.78	681.84	674.57	674.94
BIC	754.86	713.65	713.08	715.18	724.92
Lo–Mendell–Rubin Adjusted LTR	N/A	0.00	0.10	0.10	0.29
BLRT	N/A	0.00	0.00	0.04	0.44
Entropy	N/A	0.72	0.70	0.80	0.81

*Note.* AIC = Akaike information criteria; BIC = Bayesian information criterion; LRT: Likelihood Ratio Test; BLRT = Bootstrap Likelihood Ratio Test.

#### Profile differences in sympathy and prosocial behavior

Finally, we tested our last research aim of identifying sources of variance in profiles of compassion. We first regressed our outcome variables (i.e., child- and caregiver-reported sympathy and caregiver-reported prosocial behavior) on our control variables (age, gender, and caregiver education). Regarding our controls, gender significantly related to both variables, such that girls were rated higher than boys on child-reported sympathy, *b* = −0.15, *SE* = 0.06, *p* < .01, caregiver-reported sympathy, *b* = −.32, *SE* = 0.10, *p* < .01, and caregiver-reported prosocial behavior, *b* = −0.28, *SE* = 0.07, *p* < .001. Caregiver education was also negatively related to prosocial behavior, *b* = −0.09, *SE* = 0.04, *p* < .05.

Next, mean-level group differences were tested between the compassion-oriented versus non-compassion-oriented groups on our kindness variables. The two groups did not differ in neither child- nor caregiver-reported dispositional sympathy (*M*_*diff*_ = 0.03, *M*_*diff*_ = 0.06, respectively); however, the compassion-oriented group had higher levels of dispositional prosocial behavior compared to the non-compassion-oriented group (*M*_*diff*_ = 0.23, *p* < .01).

## Discussion

This study explored children’s and adolescents’ compassion using both qualitative and quantitative methods—extending previous research that has focused solely on behavioral or single-method investigations. First, how children and adolescents make meaning of their experiences of compassion in their everyday life was explored. Next, children’s and adolescents’ potential for experiencing global compassion was investigated and we tested whether children with different capacities for global compassion varied in their kind orientations.

Within youths’ compassion narratives, the target of their compassion was often a peer or a relative. This is not surprising as youth spend most of their time with peers and their family and thus have more opportunities to experience compassion toward these targets (Rubin et al., 2015). Children and adolescents may also more readily access memories about times they felt compassion for individuals within their immediate group; however, if children provided more than one narrative, it is possible that out-group targets may have been mentioned more frequently. Nevertheless, this finding shows that familial compassion is most readily experienced by children and adolescents and more research is required to assess how children and youth make meaning of their experiences of global compassion.

Previous research has almost exclusively focused on examining compassion in contexts involving physical harm (see Stellar & Keltner, 2014 for a review); however, results from youths’ narratives showed that children consider both physical and psychological/emotional harm when recalling times they experienced compassion (see Stellar et al., 2020 for similar conclusions). This underscores the versatility of compassion toward others’ suffering such that it may not matter what type of harm an individual endures, but rather how it affects their general well-being. Further exploring the contextual, affective, and behavioral markers that are related to compassion following physical and psychological harm may provide insight into potential functional differences in compassion following these types of harm (Stellar et al., 2020).

Finally, we found that half the participants mentioned helping (or wanting to help) the suffering target in their narratives. This response may reflect the prosocial commitment involved within experiences of compassion (Goetz et al., 2010). A portion of youth also mentioned comforting the target of their compassion, which likely reflects their desire to support the sufferer and ensure that their circumstance is understood. These differential responses to the suffering of others may depend on which type of suffering the other experienced, such that comforting may be more beneficial for alleviating emotional pain and direct helping may be more effective in alleviating physical pain. Further evidence is required to support these speculations.

Next, using insight from our qualitative investigation, we aimed to shed light on how youth may experience global compassion by employing vignettes about harm to peer victimizers. Promoting global compassion may help diminish us/them thinking such that if we practice extending compassion toward others, irrespective of our differences or group membership, we may eventually be able to adopt a “generalized” prosocial orientation (Ekman & Ekman, 2017). Our findings revealed that youth of all ages showed similar levels of compassion—particularly in their desire to help the harmed victimizer. Children show empathy, concern, and needs-oriented prosocial behavior early on in development (e.g., Zahn-Waxler et al., 1992) and display rudimentary forms of compassionate responding in early childhood (e.g., Green et al., 2018); thus, these capacities likely lay the foundation for experiences of global compassion starting in middle to late childhood. Further, during this age window, children advance in their cognitive and social-emotional skills such as the ability to understand multiple perspectives and also have internalized ethical principles such as fairness and care which may further aid in encouraging experiences of global compassion (Malti & Krettenauer, 2013; Spinrad & Eisenberg, 2017). This theorizing is in line with research suggesting that children are better able to exercise acceptance beginning in middle childhood (Denham et al., 2005)—a skill that likely plays a role in youths’ ability to extend their compassion toward strangers or disagreeable others. Nevertheless, further research is warranted to investigate how global compassion may unfold alongside the development of social-emotional capacities from early to middle childhood.

Regarding developmental differences, contrary to related research (i.e., Eisenberg, 1983), 15-year-olds reported less concern toward suffering victimizers compared to 11-year-olds and marginally less than 7-year-olds. This decline may be due, in part, to adolescents’ increased sensitivity toward maintaining positive group identities (LaFontana & Cillessen, 2010; Rutland et al., 2010) such that they may have preferred to emotionally distance themselves from the victimizer’s negative behavior to preserve their own positive self-image. Although more advanced forms of perspective taking develop from childhood to adolescence (Eisenberg et al., 2005; Martin et al., 2008), adolescents’ ability to apply these perspective taking skills within contexts involving the “deserved” harm of another may be thwarted when their trust and social identity is damaged. Indeed, Fett and colleagues (2014) found adolescents (13–18 years of age) who had high perspective taking skills were more sensitive to the intentions of unfair others—a sensitivity that may in turn prompt a desire for punishment or retribution (see Singer et al., 2006). Other work shows that some adolescents may use their advanced perspective taking skills to secure social status by engaging in social aggression (Yeager et al., 2015). This speaks to the necessity of developmentally tailored interventions that target a combination of social-emotional skills (e.g., perspective taking, concern, and tolerance) to effectively promote prosocial behavior (and decrease aggression; see further discussion in “Implications” section).

Next, we assessed whether there were subgroups of youth in our sample that demonstrated similar capacities for global compassion. We identified two distinct profiles of global compassion within our sample: (a) a compassion-oriented group (characterized by moderate to high levels of concern for a needy victimizer and desire to help) and (b) the non-compassion-oriented group (characterized by low levels of concern and desire to help). This supports recent research showing two subgroups of children that show divergent responses to social conflict situations: those who endorse revenge-goals and those who endorsed relationship-maintenance goals (McDonald & Asher, 2018). Indeed, it is possible that compassion-oriented children may be more pacifictic and less likely to endorse retaliation in social conflict situations in favor of maintaining relationships and group harmony.

We found that these groups of youth did not differ in their mean levels of dispositional sympathy, suggesting that sympathy may be *necessary* but *not sufficient* for experiences of global compassion. Although both the compassion-oriented and non-compassion-oriented groups had a similar ability to experience sympathy, the participants within the compassion-oriented group were able to extend their concern toward someone who had previously harmed them, perhaps due to their ability to exercise tolerance and forgive others’ wrongdoings. We also found that compassion-oriented children and adolescents had higher levels of dispositional prosocial behavior compared to non-compassion-oriented youth. Our findings bolster previous work in showing that compassion (or concern) is positively related to prosocial behavior (i.e., charitable giving, helping a stranger; Lim & DeSteno, 2016; Spinrad & Eisenberg, 2017). Differences in prosocial behavior between children and adolescents who expressed compassion versus those who did not may be related to differences in youths’ ability to transcend group boundaries and “do good” to those who have “done bad” (Enright & Song, 2020; Gilbert, 2010; Strauss et al., 2016). Future work would benefit from examining how youths’ social-emotional strengths and challenges impact youths’ proclivity for global compassion.

### Implications

Although it is important to promote concern for others, encouraging concern alone may not sufficient for fostering genuine kindness. This notion is reflected in studies that have assessed the effectiveness of school-based programs targeting prosocial behavior, suggesting that the most effective programs are those that target a wide range of compassion-related constructs in a tailored and developmentally sensitive manner (see Malti, Chaparro, et al., 2016). Findings from this study may contribute to informing the design of multicomponent strategies to target kindness, for example, promoting concern, tolerance, *and* a desire to help others may encourage youth to be more generous with their prosocial behavior.

One possible step in promoting global compassion is to increase youths’ tolerance (particularly the facet of non-judgment) by decreasing intergroup prejudice via direct positive contact with others who are perceived to be different from the self (e.g., in terms of race/ethnicity, socioeconomic status, interpersonal characteristics, etc.; Rutland & Killen, 2015). This is evidenced by research showing that diversity, particularly in schools, can have positive effects on children’s prosocial behavior and inclusive attitudes (Beelmann & Heinemann, 2014). Global compassion may also be fostered through exchanges that include perspective-giving and perspective-taking exercises, whereby individuals from diverse groups or those who experience interpersonal conflict engage in conversations that allow both parties to step in each other’s shoes (Bruneau & Saxe, 2012). These efforts may be best employed within adolescence because, as shown in this study, adolescents may be less inclined to feel compassionate concern when witnessing the harm of others who are disliked or part the out-group.

In addition to implications for interventions, this study speaks to the importance of examining compassion via different methods. In this study, we employed a novel vignette procedure to investigate how youth may respond to others in need that was informed by a qualitative investigation. This methodology extends previous research that has focused on self-, parent-, or teacher-reports via questionnaires in assessing compassion-related constructs in youth, particularly in adolescents (e.g., Malti, Zuffianò, et al., 2016; Masten et al., 2013). The major advantages of collecting data about youths’ emotions using vignettes are that they allow for the exploration of emotion *in context*, and allow participants to discuss, explore, and express subjective experiences (Gourlay et al., 2014). Future research work would benefit from exploring compassion using multiple methods (e.g., vignettes, observations, questionnaires), as well as varying the targets of compassion to better understand the role of relationships and intergroup membership in global compassion.

### Limitations and Future Directions

As with any study, this one is not without limitations. First, we gathered narrative data about only one instance of compassion. Future research should gather multiple instances of compassion to better understand youths’ experiences. Qualitative studies should also specifically disentangle the components of compassion to inform future theory and research on distinctions between compassion and related constructs. In addition, we used a cross-sectional sample and thus were unable to draw any conclusions about the causal nature and developmental trajectories of compassion—additional longitudinal work would help to uncover how compassion may unfold over time. Participants were also of moderate-highly educated backgrounds which limits how generalizable our findings are to children across diverse socioeconomic backgrounds. Along this vein, researchers may benefit from conducting comparison research between children of multicultural versus monocultural regions to understand the role of diversity in the development of global compassion. Next, we measured global compassion toward one target group: victimizers. While this was well justified in the context of the present investigation, future work may wish to extend this focus to include different targets. We also assessed youths’ desire to help using a sharing task. Incorporating additional measures (e.g., self-reports) to assess youths’ desire to help would aid in validating our method. Finally, conducting additional research on how social-emotional constructs such as forgiveness and tolerance relate to compassion is a promising avenue.

### Conclusion

This study investigated compassion from a developmental perspective using combined qualitative and quantitative methods. In doing so, it shed light on how children and adolescents experience compassion in their daily lives and the extent to which they may experience global compassion. This investigation also provided evidence regarding links between global compassion and kind orientations. Although it may be difficult to experience compassion for strangers, disliked others, and out-group members more broadly, global compassion is an important emotion to exercise in order to motivate individuals to take action against universal suffering and to break the cycle of retaliation and discrimination between individuals of different groups.
